# Systemic Proteome Alterations Linked to Early Stage Pancreatic Cancer in Diabetic Patients

**DOI:** 10.3390/cancers12061534

**Published:** 2020-06-11

**Authors:** Hong Peng, Sheng Pan, Yuanqing Yan, Randall E. Brand, Gloria M. Petersen, Suresh T. Chari, Lisa A. Lai, Jimmy K. Eng, Teresa A. Brentnall, Ru Chen

**Affiliations:** 1 The Brown Foundation Institute of Molecular Medicine, McGovern Medical School, University of Texas Health Science Center at Houston, Houston, TX 77030, USA; hongpeng12@gmail.com (H.P.); sheng.pan@uth.tmc.edu (S.P.); 2 Department of Integrative Biology and Pharmacology, McGovern Medical School, University of Texas Health Science Center at Houston, Houston, TX 77030, USA; 3 Department of Neurosurgery, McGovern Medical School, University of Texas Health Science Center at Houston, Houston, TX 77030, USA; yuanqing.yan@uth.tmc.edu; 4 Department of Medicine, University of Pittsburgh, Pittsburgh, PA 15261, USA; reb53@pitt.edu; 5 Department of Medicine, Mayo Clinic, Rochester, MN 55902, USA; Petersen.Gloria@mayo.edu (G.M.P.); Chari.Suresh@mayo.edu (S.T.C.); 6 Division of Gastroenterology, Department of Medicine, the University of Washington, Seattle, WA 98195, USA; LLai@medicine.washington.edu (L.A.L.); Terib@medicine.washington.edu (T.A.B.); 7 Proteomics Resource, The University of Washington, Seattle, WA 98109, USA; engj@u.washington.edu; 8 Section of Gastroenterology and Hepatology, Department of Medicine, Baylor College of Medicine, Houston, TX 77030, USA

**Keywords:** proteomics, pancreatic cancer, pancreatic ductal adenocarcinoma, diabetes, mass spectrometry, plasma, biomarker

## Abstract

Background: Diabetes is a risk factor associated with pancreatic ductal adenocarcinoma (PDAC), and new adult-onset diabetes can be an early sign of pancreatic malignancy. Development of blood-based biomarkers to identify diabetic patients who warrant imaging tests for cancer detection may represent a realistic approach to facilitate earlier diagnosis of PDAC in a risk population. Methods: A spectral library-based proteomic platform was applied to interrogate biomarker candidates in plasma samples from clinically well-defined diabetic cohorts with and without PDAC. Random forest algorithm was used for prediction model building and receiver operating characteristic (ROC) curve analysis was applied to evaluate the prediction probability of potential biomarker panels. Results: Several biomarker panels were cross-validated in the context of detection of PDAC within a diabetic background. In combination with carbohydrate antigen 19-9 (CA19-9), the panel, which consisted of apolipoprotein A-IV (APOA4), monocyte differentiation antigen CD14 (CD14), tetranectin (CLEC3B), gelsolin (GSN), histidine-rich glycoprotein (HRG), inter-alpha-trypsin inhibitor heavy chain H3 (ITIH3), plasma kallikrein (KLKB1), leucine-rich alpha-2-glycoprotein (LRG1), pigment epithelium-derived factor (SERPINF1), plasma protease C1 inhibitor (SERPING1), and metalloproteinase inhibitor 1 (TIMP1), demonstrated an area under curve (AUC) of 0.85 and a two-fold increase in detection accuracy compared to CA19-9 alone. The study further evaluated the correlations of protein candidates and their influences on the performance of biomarker panels. Conclusions: Proteomics-based multiplex biomarker panels improved the detection accuracy for diagnosis of early stage PDAC in diabetic patients.

## 1. Introduction

Pancreatic ductal adenocarcinoma (PDAC) is a highly lethal disease that represents the majority of pancreatic cancer cases. Most patients diagnosed with PDAC die within six months. For all stages combined, PDAC has the lowest five-year relative survival rate (9%) compared to other cancer types [1]. The high mortality of this disease is predominantly due to the advanced stage of the disease at the time of diagnosis and the rapid development of chemoresistance during treatment. Early detection of PDAC may markedly improve the survival rate [1,2]. When PDAC is detected at early stages as localized disease, the five-year survival rate could be improved to 22% [3]. If the tumor is surgically removed when at a size smaller than 2 cm and with no lymph node involvement (stage 1A), the five-year survival rate could be further improved to 40% [4]. Therefore, detection of PDAC at early stages could represent an effective strategy to improve the survival rate of PDAC patients. Unfortunately, while CA19-9, the current clinical biomarker for PDAC, is widely used for monitoring clinical course of PDAC treatment, it has a limited accuracy for detecting early stage disease. Efforts have been made to develop blood-based biomarkers to assist early detection of PDAC [5,6,7,8,9,10,11,12,13,14,15,16,17]. Due to the low prevalence of pancreatic cancer (the age-standardized rate (ASR) incidence was 7.6 per 100,000 people in North America [18]), whole population screening for early cancer detection is very challenging and economically unfeasible, requiring a testing method with nearly 100% specificity. 

Studies have shown that new-onset diabetes mellitus (DM) can be associated with PDAC and could be an early sign of pancreatic malignancy. Up to 80% of PDAC patients are either hyperglycemic or diabetic up to three years prior to the diagnosis of cancer [19,20]. Whether diabetes is a cause or effect of pancreatic neoplastic change is still controversial [20], yet PDAC risk is >8-fold increase in diabetics over the age of 65 [19]. This risk increases even further in the adults who have new onset diabetes for less than three years. By screening higher risk groups, such as new-onset adult diabetes, and by improving the accuracy of cancer test, the false-positive rate could be improved and the cancer screening could become cost effective. Thus, development of a blood-based proteomic signature that is highly specific to PDAC in a new-onset adult diabetic population may represent a realistic avenue for early detection of PDAC in this risk group.

In this study, we applied a spectral library-based mass spectrometric platform to investigate the systemic proteome alterations in the plasma from diabetic patients with PDAC in comparison to diabetic patients who were cancer-free. We sought to establish multiplex biomarker panels to facilitate the detection of early-stage PDAC among diabetic patients for future biomarker development.

## 2. Results

### 2.1. Analytical Platform

**Figure 1:**
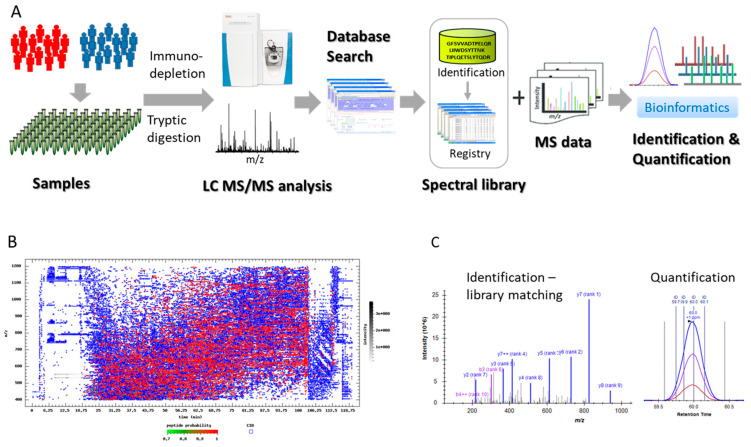
Illustration of a spectral library-based platform. (**A**) The analytical flow, (**B**) a peptide 3D map of a clinical plasma sample after depletion of the 12 most abundant proteins, (**C**) an illustration of a peptide identification and quantification using a spectral library-based approach.

A spectral library-based platform described previously was implemented for the plasma analysis in the current study [21], as illustrated in Figure 1A. To enhance the analytical sensitivity for proteins of low abundance, all plasma samples were depleted to remove the 12 most abundant proteins, including albumin, Immunoglobulin G (IgG), transferrin, fibrinogen, α1-antitrypsin, Immunoglobulin A (IgA), Immunoglobulin M (IgM), α2-macroglobulin, haptoglobin, apolipoproteins A-I and apolipoproteins A-II, and α1-acid glycoprotein. To prevent potential cross-contaminations between the clinical samples, individual spin columns were used for the plasma depletion. After the removal of the 12 most abundant proteins, the samples were processed and subjected to LC MS/MS analysis. For each analysis, about 17,000 MS/MS spectra were typically acquired and searched for the identification of peptides and proteins. A representative three-dimensional peptide map of a depleted plasma sample is demonstrated in Figure 1B. While the blue dots represent the precursors being acquired, the red dots indicate the peptides being fragmented for tandem analysis. Using Skyline software [22], a composite spectral library was constructed, which included all the peptides and proteins identified in the cohort samples with stringent criteria, and was used for peptide and protein identification in the analysis using spectral matching. This spectral library-based approach largely overcame the intrinsic caveat of data-dependent acquisition (DDA) in intermittent data acquisition, and significantly improved the data-missing issue associated with large cohort analysis using DDA-based labor-free approach. Figure 1C exemplifies the identification and quantification of a peptide using the spectral library-based platform. Each peptide was identified through spectral library by matching its fragmentation pattern and quantified using its elution profile.

A replicate experiment was carried out to assess the robustness of the platform for quantitative analysis. As shown in Figure S1, peptides with higher intensity (more abundant or/and sensitive to MS analysis) tended to have more reliable identification (small mass deviation) and quantification (smaller CV of the replicate runs). More than 85% of the peptides identified had a mass deviation ≤ 5 ppm from the theoretical values and 65% of them had a coefficient of variation (CV) ≤ 25% in the six-replicate analysis. For the peptides that were consistently identified across all replicate samples with a CV ≤ 25%, their intensities were well correlated between the replicates with an average R-squared (R^2^) = 0.98 (Figure S2).

### 2.2. Plasma Protein Candidates Associated with Early-Stage PDAC in Pilot Cohort

A pilot cohort, consisting of plasma samples from 10 diabetic patients with early-stage (stages 1 and 2) PDAC (PDAC-DM) and 10 diabetic controls (DM) (Table S1), were analyzed with over 1800 plasma proteins being interrogated. Eleven protein candidates were selected for further evaluation based on their differences in abundance between PDAC-DM versus DM groups, as well as their plasma concentration (Table S2). These proteins include apolipoprotein A-IV (APOA4), monocyte differentiation antigen CD14 (CD14), tetranectin (CLEC3B), gelsolin (GSN), histidine-rich glycoprotein (HRG), inter-alpha-trypsin inhibitor heavy chain H3 (ITIH3), plasma kallikrein (KLKB1), leucine-rich alpha-2-glycoprotein (LRG1), pigment epithelium-derived factor (SERPINF1), plasma protease C1 inhibitor (SERPING1), and metalloproteinase inhibitor 1 (TIMP1).

**Figure 2:**
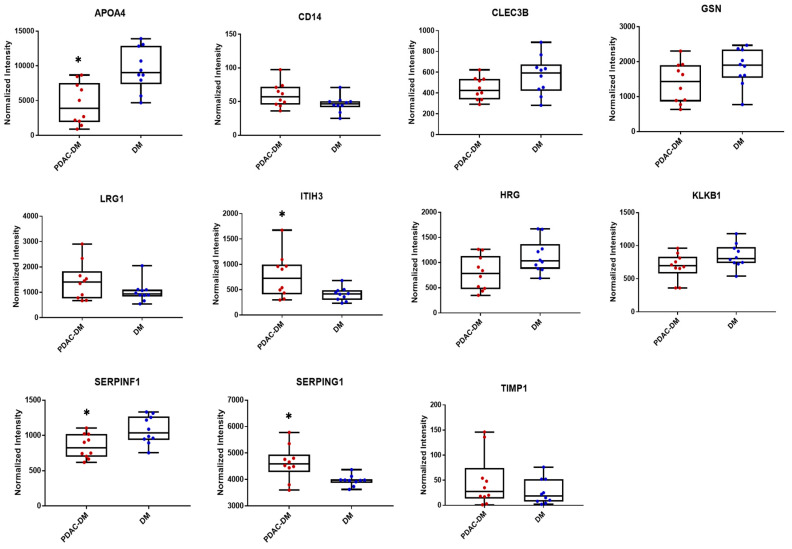
Measurements of the 11 protein candidates in the plasma samples of the pilot cohort, which includes 10 PDAC-DM (left) and 10 DM (right); * *p* ≤ 0.05.

The measurements of these proteins in the pilot cohort are illustrated in Figure 2. None of these proteins showed a significant correlation of their plasma levels with the patients’ age, gender, or duration of DM. Protein network analysis indicated that these proteins were interconnected with known PDAC pathways and oncogenes, including KRAS (GTPase KRas), SMAD4 (Mothers against decapentaplegic homolog 4), CDKN2A (Cyclin-dependent kinase inhibitor 2A), MYC (Myc proto-oncogene protein), TP53 (Cellular tumor antigen p53), TNF (Tumor necrosis factor), TGFB1 (Transforming growth factor beta-1 proprotein) and EGF (epidermal growth factor) (Figure S3). It is noteworthy that despite the relatively low blood concentration of TIMP1 (at low ng/mL level), this protein was included for further testing in this study, as its blood concentration has been previously associated with PDAC in multiple studies [8,12,13,23].

### 2.3. Analysis of the Selected Plasma Proteins in Testing Cohort

**Figure 3:**
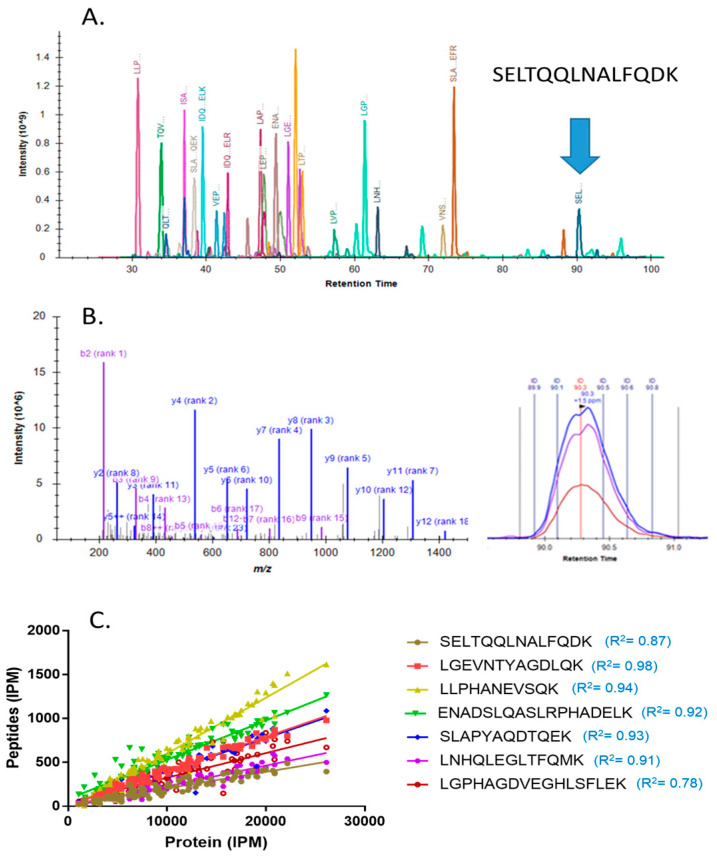
Identification and quantification of APOA4 using quantitative peptides. (**A**) Seven quantifiable peptides eluted at different retention times were selected for APOA4 quantification. The blue arrow indicates the detection of peptide SELTQQLNALFQDK, (**B**) peptide identification and quantification using SELTQQLNALFQDK as an example, (**C**) correlations of APOA4 measurement with the corresponding peptides.

The selected protein candidates were tested in a clinical plasma cohort (*N* = 99), including 50 PDAC patients with stage 1 or 2 disease and 49 controls who were cancer-free (25 chronic pancreatitis (CP) patients and 24 subjects with no pancreatic disease) (Table 1). Each protein candidate was detected and quantified with at least three unique peptides derived from the corresponding proteins. As an example, for the detection of APOA4, the intensities of seven quantifiable peptides from APOA4 eluted at different retention times were measured and used for APOA4 quantification (Figure 3A). The peptides were identified using spectral library matching and quantified based on their elution profile (Figure 3B). Across the 99 samples analyzed, the measurements of these seven peptides showed a tight correlation with APOA4 at protein level (Figure 3C).

**Figure 4:**
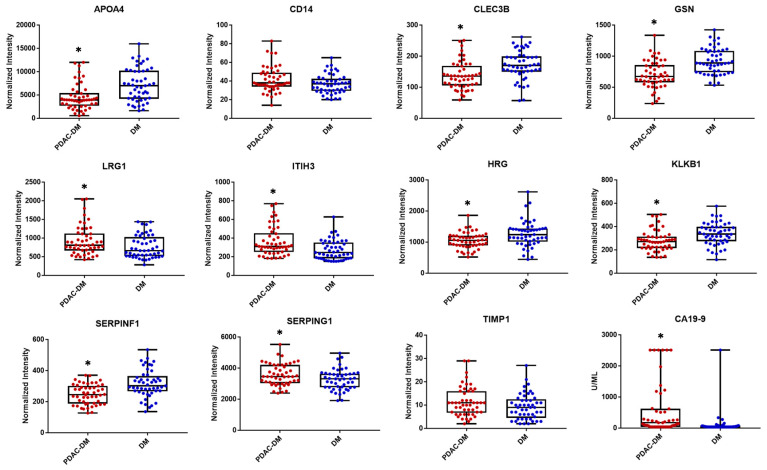
Measurements of the 11 protein candidates in the plasma samples of the testing cohort, which included 50 PDAC-DM and 49 DM controls; * *p* ≤ 0.05.

The measurements of these proteins in the plasma samples from the testing cohort are shown in Figure 4. Using receiver operating characteristic (ROC) curve analysis, the predictive performance of individual markers in distinguishing PDAC-DM from CP-DM + DM was illustrated by the area under curve (AUC) values listed in Table 2. To prevent overfitting and obtain an accurate assessment, we evaluated the prediction capacity of marker using a leave-one-out (LOO) cross-validation approach. The LOO-AUC values for these candidates as an individual biomarker ranged from 0.44 to 0.75 in separating PDAC from the control groups. As a benchmark for comparison, CA19-9 was measured in the testing cohort and had a LOO-AUC value of 0.66 in distinguishing PDAC from the control groups.

**Figure 5:**
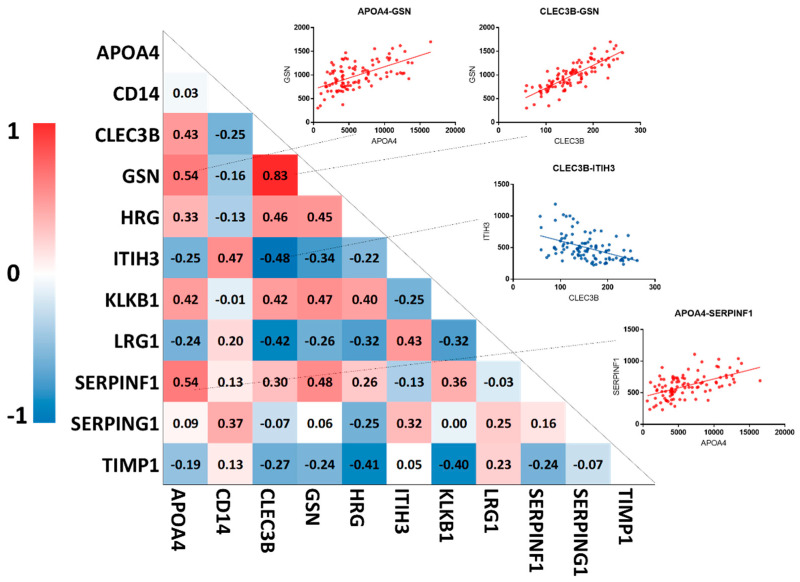
Plasma concentration correlations between the 11 protein candidates. Red indicates a positive correlation and blue indicates a negative correlation.

Further analysis of the plasma data indicated the correlation of plasma concentration of some proteins, as illustrated in Figure 5. Using a *p*-value < 0.0001 (corresponding to a Spearman r ≥ 0.40 for positive correlation or Spearman r ≤ −0.40 for negative correlation) as a cutoff for significance, the protein candidates showing positive correlation include APOA4-CLEC3B, APOA4-GSN, APOA4-KLKB1, APOA4-SERPINF1, CD14-ITIH3, GSN-CLEC3B, CLEC3B-KLKB1, GSN-HRG, GSN-KLKB1, GSN-SERPINF1, and HRG-KLKB1 and the proteins showing negative correlation include CLEC3B-ITIH3, CLEC3B-LRG1, HRG-TIMP1, and KLKB1-TIMP1. The pairs of GSN-CLEC3B (r: 0.83), APOA4-GSN (r: 0.54), and APOA4-SERPINF1 (r: 0.54) showed the most significant positive correlation, while CLEC3B-ITIH3 (r: −0.48) showed the most significant negative correlation among the candidates (Figure 5).

### 2.4. Evaluation of Multiplexed Panels

**Figure 6:**
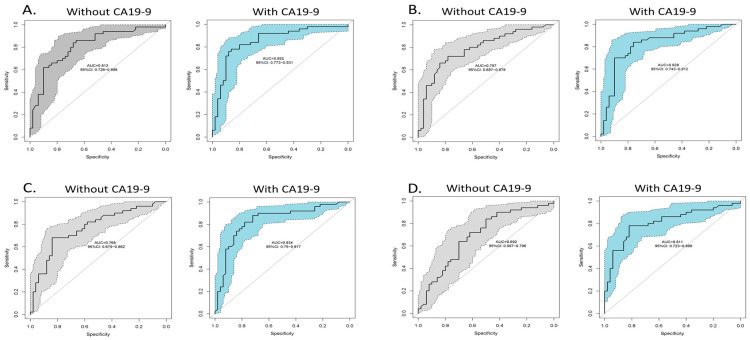
ROC analysis for the testing cohort using random forest combined with LOO approach. (**A**) Full panel, (**B**) Top-4 panel, (**C**) Correlation panel, (**D**) Non-correlation panel.

To cross-validate the predictive probability of several potential biomarker panels, a supervised learning algorithm, random forest, combined with LOO approach was applied. Using CA19-9 alone, the LOO-AUC value for discriminating PDAC-DM from the controls was 0.66 (95% confidence interval (CI): 0.54–0.78). For the full panel consisting of all 11 protein candidates (APOA4+CD14+CLEC3B+GSN+HRG+ITIH3+KLKB1+LRG1+SERPING1+SERPINF1+TIMP1), without and with the combination of CA19-9, the LOO-AUC values were 0.81 (95% CI: 0.73–0.90) and 0.85 (95% CI: 0.77–0.93), respectively (Figure 6A). Using the top four candidates with the best AUC values (APOA4+CLEC3B+GSN+SERPINF1) (Top 4 panel), the LOO-AUC values, without and with the combination of CA19-9, were 0.79 (95% CI: 0.70–0.88) and 0.83 (95% CI: 0.74–0.91), respectively (Figure 6B). In addition, based on the protein correlations indicated in Figure 5, the candidates were divided into two panels for evaluation, a Correlation panel and a Non-correlation panel. For the Correlation panel (APOA4+CLEC3B+GSN+HRG+KLKB1+SERPINF1), which included the protein candidates with a Spearman r ≥ 0.4 (*p*-value < 0.0001), the LOO-AUC values, without and with the combination of CA19-9, were 0.77 (95% CI: 0.68–0.86) and 0.83 (95% CI: 0.75–0.92), respectively (Figure 6C). For the Non-correlation panel (APOA4+ITIH3+LRG1+SERPING1+TIMP1), which included candidates with a Spearman r ≤ −0.4 (*p*-value < 0.0001), the LOO-AUC values, without and with the combination of CA19-9, were 0.69 (95% CI: 0.59–0.80) and 0.81 (95% CI: 0.72–0.90), respectively (Figure 6D). CD14 was not included in either panel, because it strongly correlated with ITIH3 (*p* < 0.0001) and SERPING1 (*p* = 0.0002), and addition of CD14 to either panel showed little influence on panel performance. The performances of the panels based on the LOO-ROC analyses are summarized in Table 3. Overall, the full panel in combination of CA19-9 outperformed other panels with a LOO-AUC of 0.85.

### 2.5. Tumor Tissue RNA Expression of the Candidates in the Cancer Genome Atlas (TCGA) Database

Using the TCGA RNA sequencing dataset available from v19.1 ProteinAtlas.org [24], the RNA expression of the 11 candidates in PDAC tissues were evaluated, and six of them, which were significantly linked to tumor stages and/or patient survival time, are illustrated in Figure S4. CLEC3B, KLKB1, and LRG1 displayed significant difference at RNA level among the tumor stages and patient survival time. While higher expression of CLEC3B and KLKB1 was associated with stage 1 and longer survival time, higher expression of LRG1 was found in stage 2 and associated with shorter survival time. Higher expression of APOA4 and SERPING1 at RNA level was significantly linked to less favorable patient survival time. ITIH3 was found with significantly higher expression in patients with late-stage diseases.

## 3. Discussion

Using a spectral library-based proteomic platform, we tested 11 protein candidates for their value in distinguishing PDAC-DM from the controls (CP-DM and DM). The panels constructed from these candidate proteins showed significant discrimination in the comparison of PDAC group and control groups, and many of the candidates have previously been implicated in PDAC or other cancers. Protein network analysis indicated that these proteins were interconnected with known PDAC pathways and oncogenes, underscoring the potential biological significances linked to their systemic changes in PDAC.

Among the protein candidates, APOA4, CLEC3B, GSN, and SERPINF1 could separate PDAC cases from controls with a LOO-AUC ≥ 0.7 as individual biomarkers. APOA4 is a major component of high-density lipoproteins and chylomicrons. Previous studies reported the association of aberrant APOA4 expression with colorectal cancer development in diabetic patients [25] and a reduced plasma abundance in colorectal cancer patients [26]. In our current study, the plasma level of APOA4 was significantly reduced in the PDAC-DM cases compared to the controls (CP-DM and DM). APOA4 is primarily expressed in the small intestine and secreted into the blood. The average RNA expression in normal pancreas tissue is almost zero according to Human Protein Atlas. TCGA data suggested that APOA4 RNA expression was not significantly different among disease stages of pancreatic cancer; however, higher APOA4 level in tumor tissue appeared to have a less favorable outcome in patient survival.

CLEC3B encodes a tetranectin protein that is designated to the extracellular region, where it binds to plasminogen, and could be involved in tissue remodeling for tumor invasion and inflammation. Aberrant expression of CLEC3B has been reported in multiple cancers, including hepatocellular carcinoma, ovarian cancer, oral squamous cell carcinoma, and lung cancer [27,28,29]. In this study, the plasma level of CLEC3B was significantly reduced in the PDAC-DM cases compared to the controls (CP-DM and DM). The reduced plasma level of CLEC3B from PDAC cases was in agreement with its tissue RNA level in TCGA database. Lower tissue RNA level of CLEC3B was associated with later stages of pancreatic cancer and less favorable outcome of cancer survival.

GSN is a calcium-dependent, actin-binding protein with two major isoforms, cytoplasmic and plasma. The cytoplasmic GSN is involved in regulating the assembly and degradation of intracellular actin filaments [30]. A previous study suggested that cytoplasmic GSN could regulate insulin secretion through remodeling the actin cytoskeleton in pancreatic β-cells [31]. On the other hand, plasma GSN is thought to be involved in the clearance of F-actin released into the circulation system by tissue or cell injury [32]. Aberrant plasma GSN is associated with various pathological conditions such as inflammation, trauma, and response to bacterial toxin. In diabetes, plasma GSN values were shown to decrease by about half in the blood of type II diabetic humans and mice models [33]. In cancer, several studies reported abnormal level of plasma GSN in multiple solid tumors [34]. Our data indicated a strong correlation of plasma GSN and CLEC3B in plasma concentration of patients with diabetes (Figure 5). The multi-functional role of GSN and its systemic changes implicated in diabetes and PDAC warrant further investigation.

Pigment epithelium-derived factor (PEDF), a serpin that has diverse biological functions, is encoded by SERPINF1. SERPINF1 plays critical roles in many physiological and pathophysiological processes, including neuroprotection, angiogenesis, fibrogenesis, and inflammation [35]. SERPINF1 regulates pancreatic vasculature development, and its deficiency causes atypical hyperplastic phenotypes in the pancreas [36]. While elevated levels of SERPINF1 have been reported in patients with diabetes and associated microvascular complications [37], a study showed that SERPINF1 was a critical negative regulator of tumor invasion in the pancreas [36]. Our data suggested that, compared to diabetic controls, the plasma level of SERPINF1 was decreased in PDAC. How the reduced plasma level of SERPINF1 contributes to the PDAC development under diabetic conditions remains to be elucidated.

Four panels generated from the 11 proteins were evaluated in the current study, including the Full panel, Top-4 panel, Correlation, and Non-correlation panels, using random forest with LOO cross-validation approach. All four panels demonstrated better performance than CA19-19 alone and showed complementary behavior with CA19-9 in distinguishing PDAC-DM from the controls. The inclusion of CA19-9 improved the performance of each panel. We also asked if the correlation of protein candidates affected biomarker panel performance. Our data suggested that aggregation of either correlated or non-correlated proteins in a biomarker panel did not significantly improve the panel performance. In the context of clinical applications, the desired sensitivity and specificity of a test is primarily determined by the prevalence of the disease. If we assume the prevalence of PDAC in adult new-onset diabetic patients is approximately ~1% [38,39], the detection accuracy of CA19-9 alone is estimated to be only 40%. With the combination of the Full panel, the detection accuracy could be improved to 80% in distinguishing PDAC patients with early-stage diseases from controls, reaching a positive predictive value (PPV) of 4%. This opens the opportunity for further development of biomarkers to assist clinical workout to rule in diabetic patients for imaging tests, such as endoscopic ultrasound (EUS), Magnetic Resonance Imaging (MRI), or computed tomography (CT). It is notable that one of the unique advantages of spectral library-based platform is its enormous multiplexing capacity, which affords detection of multiple proteins simultaneously with high specificity and is highly robust for biomarker panel detection using quantifiable peptides. In this study, our platform could measure more than 200 plasma proteins in a single analysis with accurate quantification in an automatic, high-throughput fashion.

## 4. Materials and Methods

### 4.1. Patients and Plasma Samples

The study was approved by the Institutional Review Boards at the University of Washington (6276, approved on 11 June 2019), University of Pittsburgh (MOD19070256, approved on 21 October 2019), and Mayo Clinic (356-06, approved on 25 January 2012, 354-06, approved on 8 November 2019). All subjects from the 2 pilot cohorts gave their informed consent for inclusion before they participated in the studies. The pilot cohort was from Mayo Clinic and included 10 diabetic patients with PDAC and 10 diabetic patients who were cancer-free. The demographic information of these patients is provided in Table S1. The testing cohort from University of Pittsburgh (Pittsburgh cohort) included 50 PDAC patients with early-stage disease (3-1A, 1-1B, 11-2A, 35-2B), 25 chronic pancreatitis patients, and 24 controls with no pancreatic diseases (Table 1). All these patients have diabetes. The PDAC patients were staged according to histology, imaging, and clinical assessment. The blood samples were drawn into purple-top tubes (Becton Dickinson, Franklin Lakes, NJ, USA), with EDTA (ethylenediaminetetraacetic acid) as an anticoagulant, and then centrifuged at 1200 rpm for 20 min within four hours of collection. The aliquoted plasma was stored at –80 °C until analysis.

### 4.2. Sample Preparation for Proteomic Analysis

Equal volumes (6 µL) of plasma from each patient was depleted to remove the top 12 abundant proteins using depletion spin columns (ThermoFisher Scientific, Waltham, MA, USA). The proteins were deglycosylated with PNGase F (New England Biolabs, Ipswich, MA, USA), reduced with 10 mM dithiothreitol at 50 °C for 1 h and alkylated with 25 mM iodoacetimide at room temperature in the dark for 30 min. After buffer exchange (Vivaspin^®^ 500 filter), the samples were digested with sequencing-grade modified trypsin at 1:30 ratio (weight:weight) at 37 °C for 18 h. The samples were dried down and re-suspended in 50 µL 0.1% formic acid for MS analysis.

### 4.3. LC-MS/MS Analysis

The samples were blinded and analyzed in a random order. The LC MS/MS system included a Q Exactive^TM^ Plus mass spectrometer (ThermoFisher Scientific) coupled with a nanoACQUITY HPLC (Waters, Milford, MA, USA). The peptides were first loaded onto a trapping column (100 µm × 3 cm) then separated with an analytical column (75 µm × 30 cm). The trapping column and the analytical column were packed with ProntoSIL 120 Å-5 µm-C18 AQ beads (Mac-Mod, Chadds Ford, PA, USA). The analytical column was house-made with a tip pulled with a Laser Fiber Puller P-2000 (Sutter Instruments, Novato, CA, USA) at the end of the column. The sample was loaded onto the trapping column with 98% Buffer A (0.1% formic acid in water)/2% Buffer B (0.1% formic acid in acetonitrile) at a flow rate of 2 µL/min for 10 min, and separated by a linear gradient from 5 to 30% B for 90 min, followed by flushing with 80% B for 10 min and equilibration with 2% B for 20 min. The LC gradient lasted 120 min with a flow rate of 0.3 µL/min. Electrospray ionization was operated in a positive mode at a voltage of 2.1 kV. Data-dependent acquisition (DDA) was performed on a Q Exactive^TM^ Plus mass spectrometer. The survey scan was done with 70,000 resolution at 200 m/z from 400 to 1200 m/z with an Automatic Gain Control (AGC) target of 1e6 and max injection time of 100 ms. The precursors were isolated in the quadrupole within an isolation window of 1.6 m/z. The top 50 monoisotopic masses with 2 to 4 plus charges were selected with a minimum intensity threshold of 5e4, then fragmented by higher-energy collisional dissociation (HCD). The DDA cycle time was ~3 s.

### 4.4. Data Analysis

The MS data were searched against the UniProt human protein database for peptide/protein identification using the Comet algorithm [40] embedded in the Trans-Proteomic Pipeline [41]. Carbamidomethylation of cysteine was set as fixed modification, and oxidation of methionine and deamidation of asparagine were set as variable modifications. The peptide assignment was validated with PeptideProphet [42], and a probability score ≥ 0.9 in correspondence with an FDR (false discovery rate) of 0.01 was applied to filter the peptides. The Skyline software [22] was used for quantitative analysis of the DDA data. The composite spectral library was built using all of the DDA data collected from the samples analyzed in each batch. Quantification was made at MS1 level using the sum of the first 3 monoisotopic peaks. The abundance of each peptide was normalized to total ion current (TIC) and presented as ion per million (IPM) using the following formula: Normalized Intensity (IPM) = Peptide Intensity/TIC × 1,000,000. Protein quantification was achieved by summation of the normalized intensities of the corresponding peptides.

### 4.5. CA19-9 Analysis

CA19-9 levels in plasma were measured in a randomized, blinded fashion using a commercial ELISA kit (DRG International Inc., Springfield, NJ, USA) according to manufacturer’s instructions. Ten microliters of each sample were incubated along with an assay buffer in 96-well ELISA plates precoated with murine monoclonal anti-CA19-9 antibody for 90 min at 37 °C. After washing the wells 4 times with wash buffer, a horseradish peroxidase-conjugated anti-CA19-9 was added and incubated for 90 min at 37 °C. After washing the wells 4 times with wash buffer, 100 µL of chromogen with substrate were added and incubated at room temperature in dark for 20 min. The reaction was stopped by the addition of 100 µL of stop solution and the absorbance at 450 nm was determined using a Synergy H1 Multi-Mode plate reader (BioTek, Winooski, VT, USA) within 15 min of addition of stop solution. Results were mean absorbance of duplicate wells. The subjects with a CA19-9 reading < 37.5 may possibly have included patients who lack the Lewis antigen A.

### 4.6. Statistical Analysis

In this study, the supervised random forest algorithm was used to build the prediction model. Random forest is an ensemble learning method by collecting multiple decision trees and aggregating the results into one final output. To avoid the model overfitting and provide an accurate assessment, leave-one-out approach was used. Predication capacity of markers was evaluated by the receiver operating characteristic (ROC) curve and the area-under-curve (AUC) value. All statistical analysis was conducted in R (version 3.5.3). A *p*-value ≤ 0.05 was considered as statistical significance. The protein correlation analysis was computed using nonparametric Spearman correlation with 95% confidence interval. A two-tail *p*-value ≤ 0.0001 was considered statistical significance.

## 5. Conclusions

Diabetes is a risk factor for PDAC. Detection of PDAC among diabetic patients, especially adult new-onset diabetics, may represent a practical avenue to facilitate early diagnosis of PDAC in this risk population. This study provided novel empirical data to reveal the systemic proteome alterations linked to early-stage PDAC within the diabetic background. While the study was not mechanistically driven, the changes of plasma proteome associated with PDAC-DM may provide useful clues to elucidate the molecular events and interplays between the two diseases. Furthermore, using a spectral library-based proteomic approach, a roster of protein candidates was evaluated based on their plasma level in the context of development of surrogate biomarker panels to assist PDAC diagnosis.

## Figures and Tables

**Table 1:** Summary of sample sets.

		Pilot Cohort	Testing Cohort
Total samples		20	99
PDAC		10	50
Stage	Ia	1	3
	Ib		1
	IIa	9	11
	IIb		35
	III		0
	VI		0
Control		10	49
Normal Imaging			11
Chronic pancreatitis			25
Healthy			3
Normal pancreas			8
Other benign conditions			2
Diabetes	Yes	20	99
	No	0	0

**Table 2:** AUC of protein candidates in the testing cohort.

Gene	Protein Name	Fitting AUC *	LOO AUC **
APOA4	Apolipoprotein A-IV	0.72	0.69
CD14	Monocyte differentiation antigen CD14	0.60	0.44
CLEC3B	Tetranectin	0.72	0.71
GSN	Gelsolin	0.77	0.75
HRG	Histidine-rich glycoprotein	0.68	0.66
ITIH3	Inter-alpha-trypsin inhibitor heavy chain H3	0.69	0.66
KLKB1	Plasma kallikrein	0.69	0.67
LRG1	Leucine-rich alpha-2-glycoprotein	0.63	0.59
SERPINF1	Pigment epithelium-derived factor	0.73	0.71
SERPING1	Plasma protease C1 inhibitor	0.62	0.59
TIMP1	metalloproteinase inhibitor 1	0.60	0.56
CA19.9		0.77	0.66

* Fitting AUC—AUC obtained from training data; ** LOO AUC—Leave-One-Out AUC.

**Table 3:** Summary of LOO-ROC analysis on biomarker panels.

	Full Panel		Top 4 with Highest LOO AUC		Correlation Panel		Non-Correlation Panel		CA19-9
Panel	APOA4+CD14+CLEC3B+GSN+HRG+ITIH3+KLKB1+LRG1+SERPING1+SERPINF1+TIMP1		APOA4+CLEC3B+GSN+SERPINF1		APOA4+CLEC3B+GSN+HRG+KLKB1+SERPINF1		APOA4+ITIH3+LRG1+SERPING1+TIMP1		CA19-9
	w/o CA19-9	w CA19-9	w/o CA19-9	w CA19-9	w/o CA19-9	w CA19-9	w/o CA19-9	w CA19-9	CA19-9
LOO AUC (95% CI)	0.81 (0.73–0.90)	0.85 (0.77–0.93)	0.79 (0.70–0.88)	0.83 (0.74–0.91)	0.77 (0.68–0.86)	0.83 (0.75–0.92)	0.69 (0.59–0.80)	0.81 (0.72–0.90)	0.66 (0.54–0.78)
Sensitivity—True positive rate (TPR)	0.76	0.80	0.78	0.80	0.74	0.82	0.66	0.82	0.94
Specificity—True negative rate (TNR)	0.70	0.80	0.68	0.74	0.68	0.76	0.64	0.72	0.40

## Supplementary Materials

The following are available online at https://www.mdpi.com/2072-6694/12/6/1534/s1. Figure S1: Replicate evaluation of proteomic plasma data, Figure S2: Correlation of replicate analysis in peptide intensity, Figure S3: Protein interaction of APOA4, CD14, CLEC3B, GSN, HRG, ITIH3, KLKB1, LRG1, SERPING1, SERPINF1, and TIMP1 with known oncogenes associated with PDAC, Figure S4: Tissue RNA expression analysis using TCGA database, Table S1: The demographic information for the pilot cohort, Table S2: Selected protein candidates in pilot cohort.
