# Supplementary material for: Systemic Proteome Alterations Linked to Early Stage Pancreatic Cancer in Diabetic Patients
Source: Cancers (Basel). 2020 Jun 11;12(6):1534. doi: 10.3390/cancers12061534 (PMC7352938; doi:10.3390/cancers12061534)
Supplement: Supplementary file 1 [file cancers-12-01534-s001.pdf]

Systemic Proteome Alterations Linked to Early Stage Pancreatic Cancer in Diabetic Patients

Hong Peng, Sheng Pan, Yuanqing Yan, Randall E. Brand, Gloria M. Petersen, Suresh T. Chari, Lisa A. Lai, Jimmy K. Eng, Teresa A. Brentnall and Ru Chen

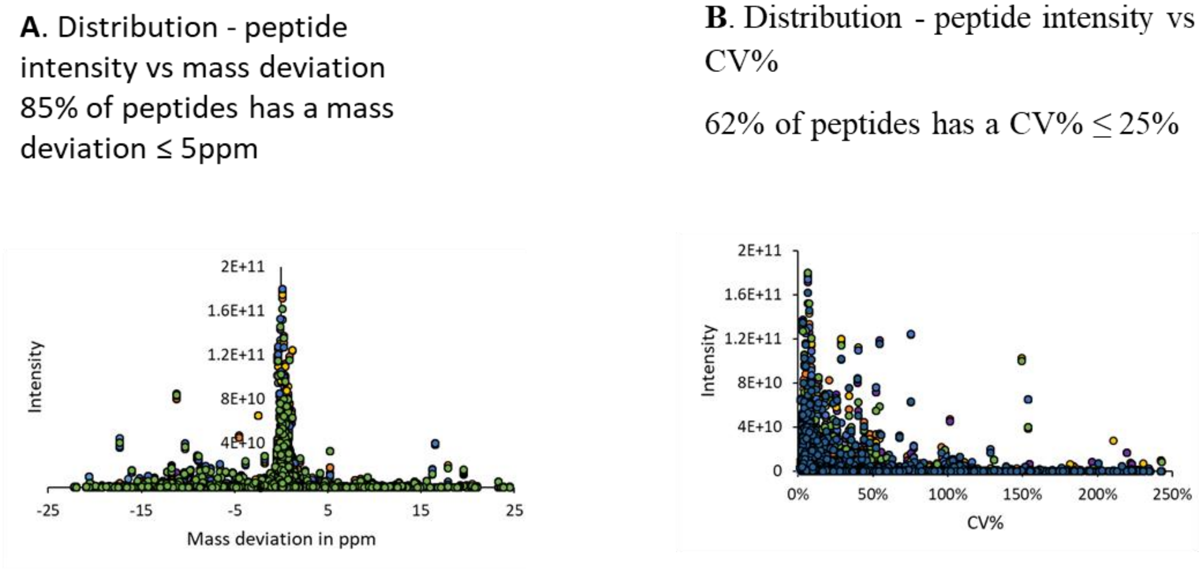

Figure S1. Replicate evaluation of proteomic plasma data. A) Distribution of peptide intensity versus mass deviation; B) Distribution of peptide intensity versus CV%.

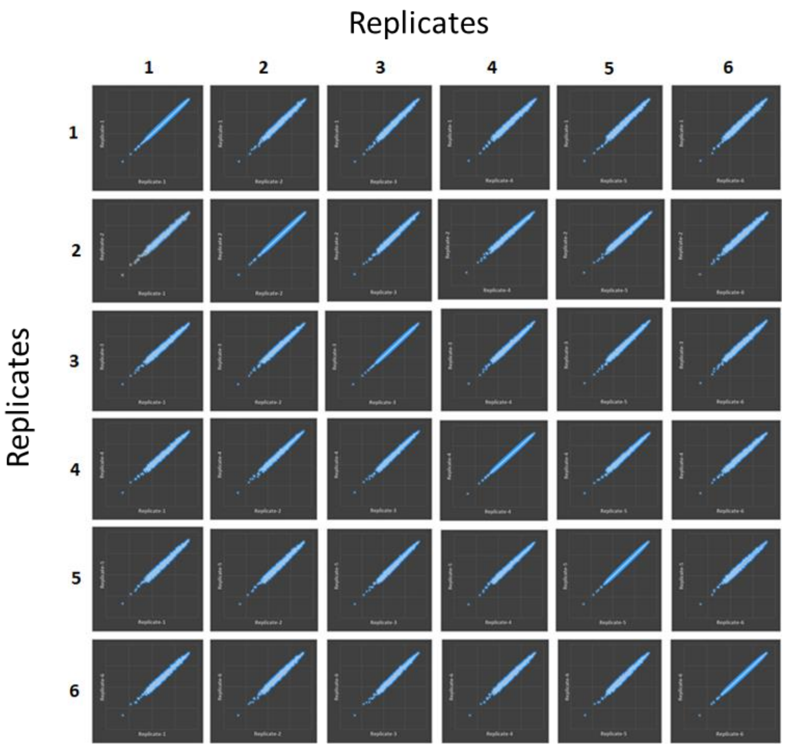

Figure S2. Correlation of replicate analysis in peptide intensity.

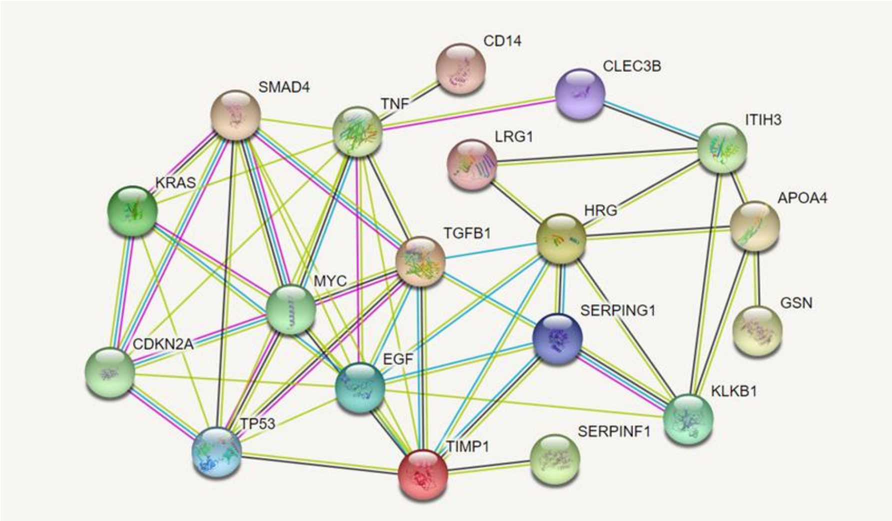

Figure S3. Protein interaction of APOA4, CD14, CLEC3B, GSN, HRG, ITIH3, KLKB1, LRG1, SERPING1, SERPINF1 and TIMP1 with known oncogenes associated with PDAC.

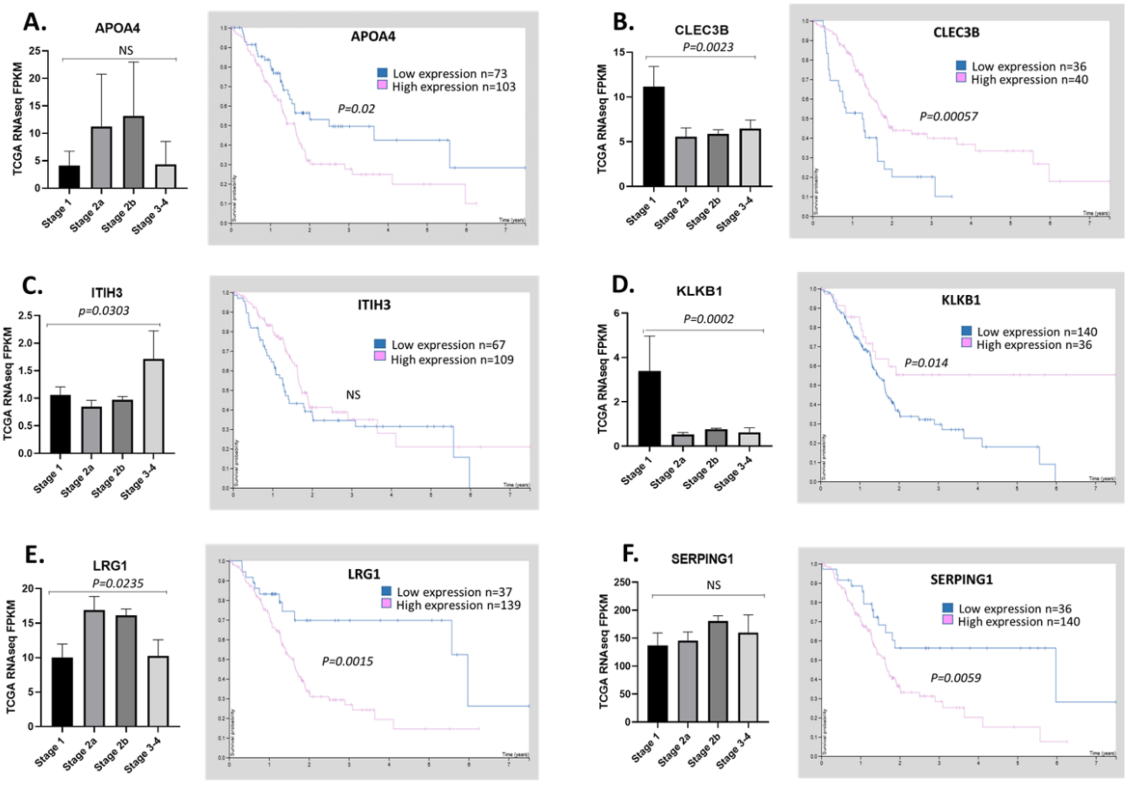

Figure S4. Tissue RNA expression analysis using TCGA database. **A**) APOA4, **B**) CLEC3B, **C**) ITIH3, **D**) KLKB1, **E**) LRG1, **F**) SERPING1.

Table S1. The demographic information for the pilot cohort.

Table S1. The demographic information for the pilot cohort.

Disease group	DM	Age	Sex	DM duration (year)	PDAC stage
Ctrl DM	Yes	79	M	6	
Ctrl DM	Yes	65	F	2	
Ctrl DM	Yes	63	M	4	
Ctrl DM	Yes	74	M	21	
Ctrl DM	Yes	63	M	9	
Ctrl DM	Yes	80	M	15	
Ctrl DM	Yes	74	M	4	
Ctrl DM	Yes	76	F	41	
Ctrl DM	Yes	76	M	3	
Ctrl DM	Yes	74	M	14	
PDAC DM	Yes	70	M	12	2A
PDAC DM	Yes	62	M	8	2
PDAC DM	Yes	75	M	6	2B
PDAC DM	Yes	81	M	7	2B
PDAC DM	Yes	65	M	22	2
PDAC DM	Yes	74	M	3	2B
PDAC DM	Yes	65	F	5	2
PDAC DM	Yes	77	M	10	1
PDAC DM	Yes	75	F	4	2
PDAC DM	Yes	77	M	2	2B

Table S2. Selected protein candidates in pilot cohort.

Table S2. Selected protein candidates in pilot cohort

Gene name	Protein name	Plasma conc. range (ng/ml) Ref: Mol Cell Proteomics. 2011 10(9):M110.006353	Fitting AUC
APOA4	Apolipoprotein A-IV	240000	0.89
CD14	Monocyte differentiation antigen CD14	420	0.72
CLEC3B	Tetranectin	8200	0.72
GSN	Gelsolin	300000	0.72
HRG	Histidine-rich glycoprotein	100000	0.75
ITIH3	Inter-alpha-trypsin inhibitor heavy chain H3	150000	0.78
KLKB1	Plasma kallikrein	2600	0.74
LRG1	Leucine-rich alpha-2-glycoprotein	20000	0.67
SERPING1	Plasma protease C1 inhibitor	280000	0.82
SERPINF1	Pigment epithelium-derived factor	5000	0.80
TIMP1	metalloproteinase inhibitor 1	14	0.60
